# Comparative Evaluation of Tear Strength and Tensile Strength of Different Types of Gingival Mask Materials: An In Vitro Study

**DOI:** 10.7759/cureus.67944

**Published:** 2024-08-27

**Authors:** Zalak Raval, Kalpesh Vaishnav, Twinkle Sanghani, Shaiva Thakar, Ruchi Patel, Ravi Joshi, Sanidhya Makwana, Jinsa A Yohannan

**Affiliations:** 1 Prosthodontics, Crown and Bridge, Karnavati School of Dentistry, Gandhinagar, IND; 2 Dental Assistant, Marcelino Dental Arts, Little Silver, USA; 3 Healthcare and Hospital Management, California State University, San Bernardino, USA; 4 Dental Surgery, Karnavati School of Dentistry, Gandhinagar, IND

**Keywords:** soft tissue cast, implants, peri-implant soft tissue, esthetics, accuracy

## Abstract

Introduction: The implant-supported prosthetic treatment strategy is commonly chosen in modern dentistry to address tooth loss caused by a variety of conditions or dental defects. To achieve healthy and natural-looking results in implant dentistry, it is essential to replicate the peri-implant soft tissue. The gingival tissue that surrounds implants is quite accurately replicated by gingival masks. They facilitate more accurate prosthesis restoration design, enhance periodontal health, and promote oral cleanliness. Furthermore, gingival masks allow for the accurate observation of superstructure seating on implant analogs, which is essential for creating superstructures that fit perfectly.

Aim and objective: To evaluate the change in tear strength and tensile strength of three different gingival mask materials (esthetic mask auto mix, Gi-Mask and Gingifast Rigid) available in the market at various time intervals.

Materials and methods: Total of 540 specimens were fabricated with 180 samples of each group. Changes in tensile strength and tear strength of three different gingival mask materials (esthetic mask auto mix, Gi-Mask and Gingifast Rigid) at intervals of one day, three days, and seven days were measured by a universal testing machine. Statistical analysis was done using one-way ANOVA and Tukey Post Hoc test. We also performed correlation and regression analyses on tear and tensile strength.

Results: The null hypothesis, which is supported by these data, claims that there is no discernible variation in the tear strength and tensile strength of three distinct materials across various time intervals. Thus, the null hypothesis was rejected, and it was concluded that there was a significant change in the tear strength and tensile strength of these gingival mask materials at different time intervals. Esthetic mask auto mix has a high tear strength compared to Gi-Mask and gingifast rigid. Gi-Mask has the least tear strength among all three. Tensile strength decreases as time increases, but the Esthetic mask auto mix has high strength compared to Gi-Mask and gingifast rigid.

Conclusion: Selecting the right material for gingival masks is essential, taking into account the clinical scenario and the articulation time. Time influences gingival mask materials' tear strength and tensile strength, which impacts their performance and durability. Esthetic mask auto mix has a high tear and tensile strength compared to Gi-Mask and gingifast rigid.

## Introduction

The implant-supported prosthetic treatment strategy is commonly chosen in modern dentistry to address tooth loss caused by a variety of conditions or dental defects [1]. With the development of biocompatible materials and technology in recent years, implant-supported prostheses for tooth loss have shown promise in terms of long-term success through several research projects in the literature [2]. Research has revealed that a lack of passive fit can result in a number of challenges including longevity of dental implant treatment and increased stress or strain on the prosthetic components, implants, or peri-implant bone [3]. Appropriate impression techniques must be used to accomplish this match between the restoration and the supporting tissues, and accuracy in master model creation during restoration production processes becomes essential.

In implant dentistry, emulating the soft tissue surrounding an implant analog is essential to producing outcomes that seem healthy and natural. In order to achieve better conformity of the prosthetic restoration made to the patient’s gum and esthetics, the patient’s gingival form should be reconstructed; to do this, soft material is used to let the dentist have the patient’s gingival form in the laboratory. Gingival masks are extremely accurate replicas of the gingival tissue surrounding implants that help with improved periodontal health, better oral cleanliness, and more precise prosthetic restoration design [4]. Furthermore, gingival masks provide the observation of accurate superstructure seating on implant analogs and are essential in creating superstructures that fit perfectly.

A number of materials, including elastomeric impression or soft-liner materials, have been proposed to simulate soft tissues in the master models made during the traditional laboratory fabrication of tooth or implant-supported restorations, in response to the increase in implant-supported restorations [5]. Nonetheless, manufacturers are increasingly using different physical and chemical gingival masks made especially for this purpose, for reasons such as the difficulty of application or adherence to similar chemical qualities of the imprint surface. Usually used around the analog in implant impressions, the gingival mask makes it possible to fabricate the restoration during fixed prosthesis fabrication stages in a way that is appropriate for the soft tissue in contact with the restoration. This means that the restoration can be taken out and repositioned on the implant several times without compromising the emergence profile or putting wear and deformation on the master model [6]. It should be remembered, nevertheless, that the alcohol utilized in these materials as a plasticizer may evaporate as a result of polymerization, changing the dimensions.

Understanding the properties of gingival masks, such as tear strength and tensile strength over time, can help us use this material more effectively. Currently, there is limited research on these characteristics of various gingival masks. Therefore, this study aims to compare the tensile strength and tear strength of three different gingival mask materials (esthetic mask auto mix, Gi-Mask and Gingifast Rigid) at different time intervals.

## Materials and methods

This in vitro study was conducted at the Department of Prosthodontics, Karnavati School of Dentistry, Gandhinagar, Gujarat, for seven days in April 2024. Ethical clearance was not required since the study did not involve human samples. This in vitro study aimed to assess the tensile and tear strength of three different gingival mask materials (Group A is esthetic mask auto mix, Group B is Gi-Mask and Group C is Gingifast Rigid) at intervals of one day, three days, and seven days.

Preparation of stainless-steel die for tear and tensile strength

**Figure 1:**
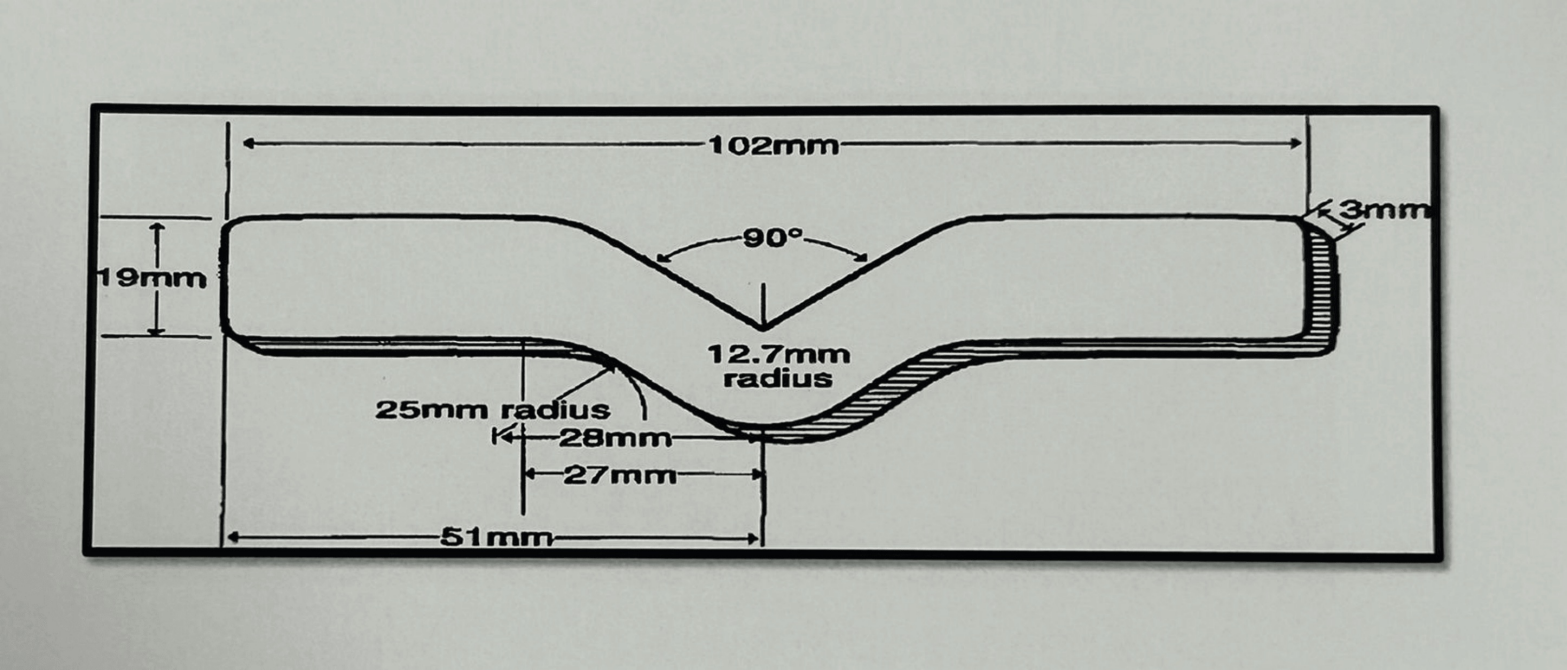
Schematic diagram of trouser-shaped specimen Figure credit: Dr. Zalak Raval

**Figure 2:**
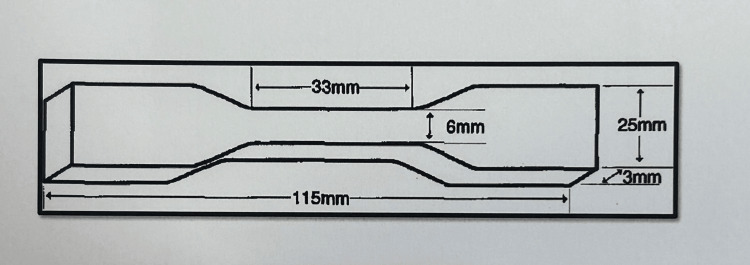
Schematic diagram of dumbbell-shaped specimen Figure credit: Dr. Zalak Raval

**Figure 3:**
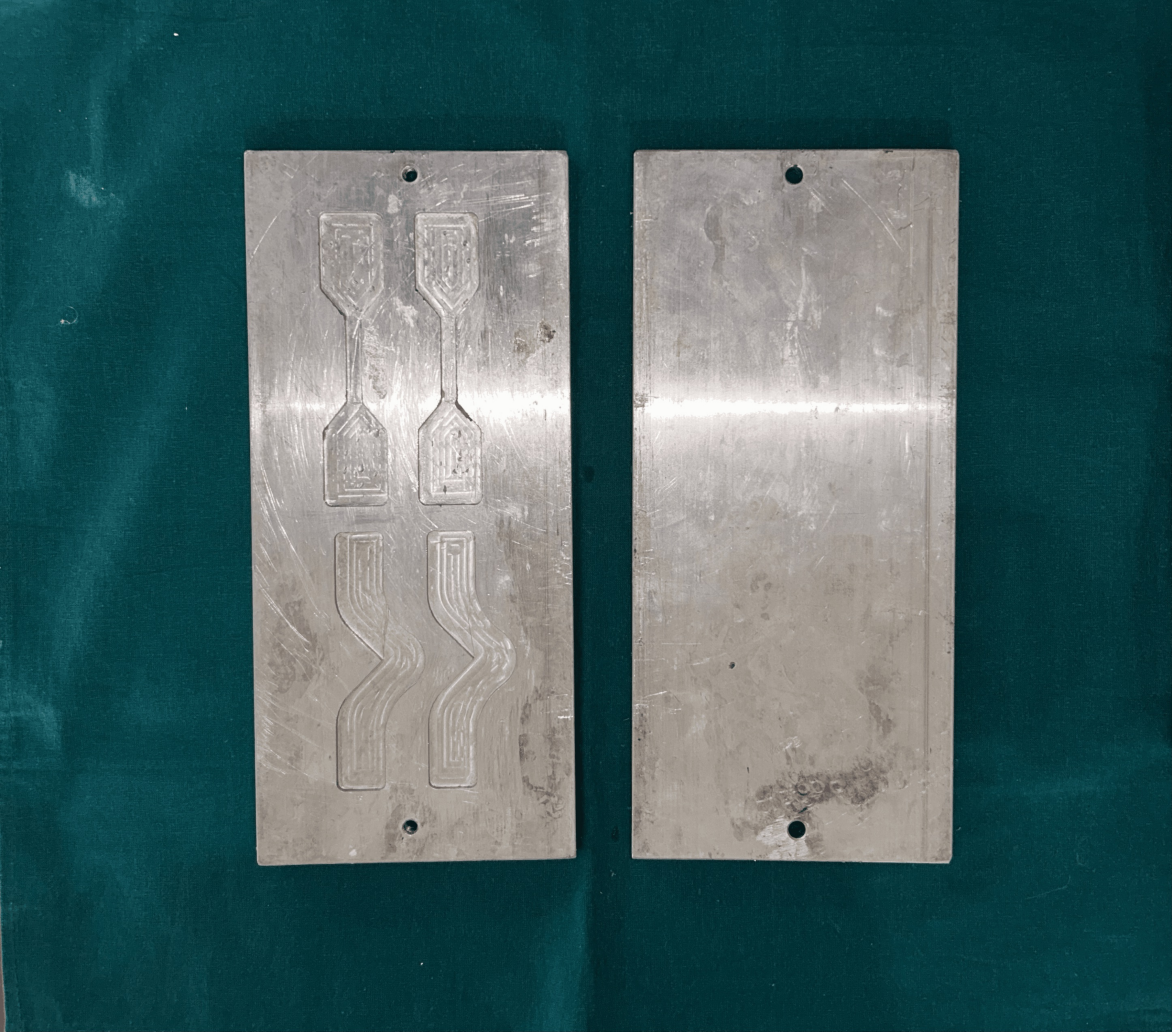
Metal die (ASTM specifications D142 and D624) Figure credit: Dr. Zalak Raval ASTM: American Society for Testing and Materials

With the use of computer-aided design and computer-aided manufacturing (CAD-CAM) design, a metal die was made to create the specimen. This included two patterns, one in the form of a trouser shape (Figure 1) and the other in the form of a dumbbell shape (Figure 2). It was created in accordance with American Society for Testing and Materials (ASTM) specifications D142 and D624 (Figure 3).

Preparation of gingival mask specimens

A total of 540 gingival mask specimens were created in precise accordance with the manufacturer's instructions and the ASTM D624 and D142 specifications. The materials were manipulated as per manufacturer's instructions and spread over the entire die surface. The samples were carefully taken out after setting of material, and flash was removed. After removal of each specimen, die was cleaned with alcohol and air dried to ensure die was totally free of any surface contaminants before making a new specimen. Specimens were stored at room temperature.

Study design 

**Figure 4:**
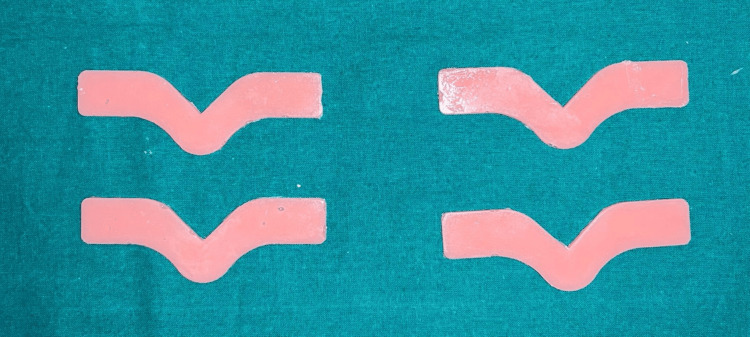
Group A-Tensile strength specimens Figure credit: Dr. Zalak Raval

**Figure 5:**
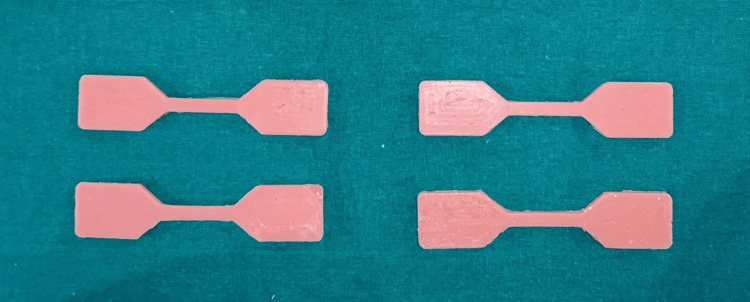
Group A-Tear strength specimens Figure credit: Dr. Zalak Raval

**Figure 6:**
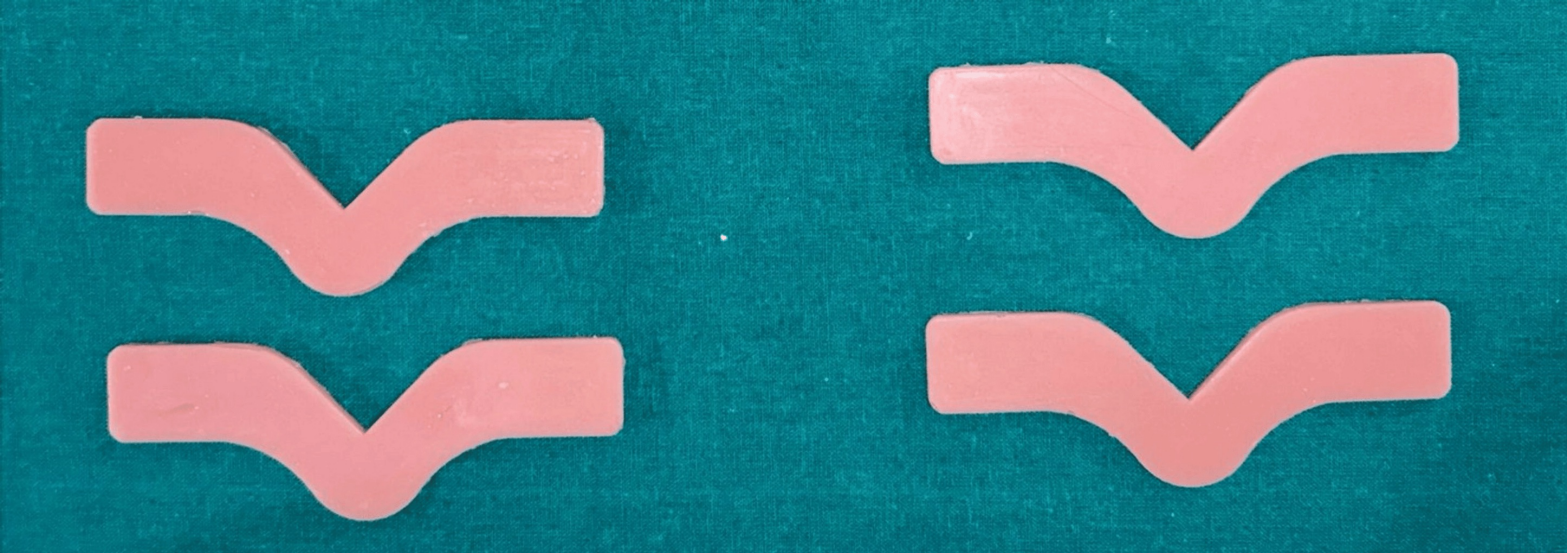
Group B-Tensile strength specimens Figure credit: Dr. Zalak Raval

**Figure 7:**
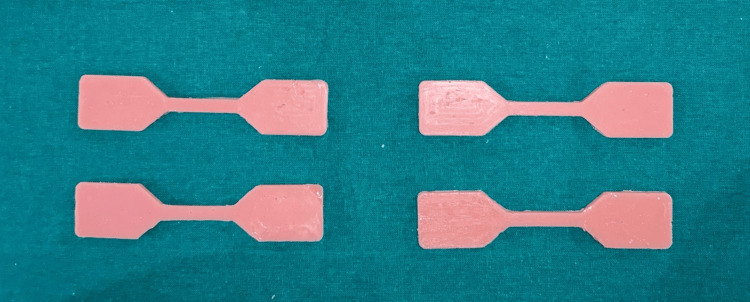
Group B-Tear strength specimens Figure credit: Dr. Zalak Raval

**Figure 8:**
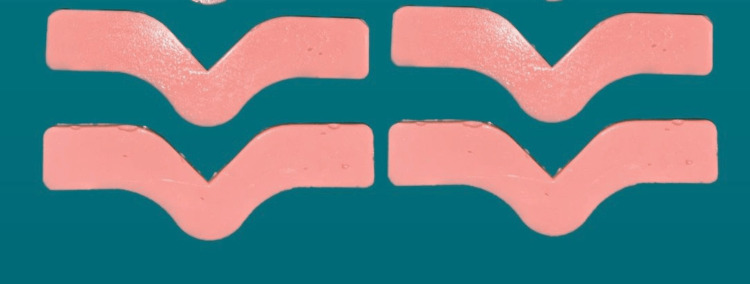
Group C-Tensile strength specimens Figure credit: Dr. Zalak Raval

**Figure 9:**
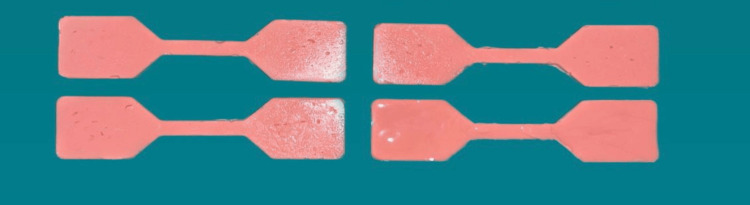
Group C-Tear strength specimens Figure credit: Dr. Zalak Raval

A total of 540 specimens were fabricated with 180 samples of each group, 90 for tear strength and 90 for tensile strength. Specimens were named as TS and DS based on the shape. 270 TS specimens were used to check tear strength, and 270 DS specimens were used to check the tensile strength. Group A is esthetic mask auto mix, Group B is Gi-Mask and Group C is Gingifast Rigid (Figure 4-9).

Evaluation of tear strength and tensile strength

Using the universal testing machine (Instron 33R 4467), the tear strength and the tensile strength of each specimen were assessed. To test the tear strength, a trouser-shaped specimen was measured at three different locations near its centre. Then, it was kept in the grasp of a universal testing machine and stretched until it ruptured at the rate of 10 mm per minute. Software was used to record the amount of force required to break the specimen.

The equation used to calculate the tear strength is T=F/D. Here F is the maximum force and D is the thickness of the specimen. Dumbbell-shaped specimens were put under tension in the grasp of a universal testing machine to determine the tensile strength. Then, the upper member was moved at a speed of 500 mm per minute, and the maximum force before breaking and elongation was noted.

The equation used to calculate the tensile strength is T=F/A. Here F is a force of magnitude required before breaking and A is the cross-sectional area of the unstrained specimen.

## Results

A total of 540 specimens were prepared. The observer in this study was blinded to the results. The change in values of tear strength and tensile strength were analyzed and inter-group and intra-group data were obtained. That data was analyzed statistically by using one-way ANOVA and Tukey Post Hoc test.

In the present study, comparison of tear strength of Group A, Group B, and Group C at a time interval of one day, three days, and seven days was done using one-way ANOVA and Tukey Post Hoc test. Based on the results we can say that tear strength decreases as time increases except Group A in which tear strength increases as time interval increases (Tables 1, 2).

**Table 1:** Comparison of tear strength in between groups and within groups by one-way ANOVA df: Degree of freedom; S: Significant

		Sum of squares	df	Mean Square	F	ANOVA P-Value	Significance
Day 1	Between Groups	25.6347	2	12.8173	63.3807	<0.00001	S
	Within Groups	5.4601	27	0.2022			
	Total	31.0948	29				
Day 3	Between Groups	777.464	2	388.732	329.6781	<0.00001	S
	Within Groups	31.8364	27	1.1791			
	Total	809.3004	29				
Day 7	Between Groups	953.954	2	476.977	154.25795	<0.00001	S
	Within Groups	83.486	27	3.0921			
	Total	1037.44	29				

**Table 2:** Summary of Tukey Post Hoc test to compare tear strength of three gingival mask materials with each other at different time intervals *: The mean difference is significant at the 0.05 level

	(I) Group	(J) Group	Mean Difference (I-J)	Std. Error	Sig.	95% Confidence Interval	
						Lower Bound	Upper Bound
Day 1	GROUP A	GROUP B	1.62000^*^	0.20112	.000	1.1213	2.1187
		GROUP C	-0.56000^*^	0.20112	.025	-1.0587	-.0613
	GROUP B	GROUP A	-1.62000^*^	0.20112	.000	-2.1187	-1.1213
		GROUP C	-2.18000^*^	0.20112	.000	-2.6787	-1.6813
	GROUP C	GROUP A	0.56000^*^	0.20112	.025	.0613	1.0587
		GROUP B	2.18000^*^	0.20112	.000	1.6813	2.6787
Day 3	GROUP A	GROUP B	12.40000*	0.48562	.000	11.1959	13.6041
		GROUP C	7.34000*	0.48562	.000	6.1359	8.5441
	GROUP B	GROUP A	-12.40000*	0.48562	.000	-13.6041	-11.1959
		GROUP C	-5.06000*	0.48562	.000	-6.2641	-3.8559
	GROUP C	GROUP A	-7.34000*	0.48562	.000	-8.5441	-6.1359
		GROUP B	5.06000*	0.48562	.000	3.8559	6.2641
Day 7	GROUP A	GROUP B	13.21000*	0.78639	.000	11.2602	15.1598
		GROUP C	10.10000*	0.78639	.000	8.1502	12.0498
	GROUP B	GROUP A	-13.21000*	0.78639	.000	-15.1598	-11.2602
		GROUP C	-3.11000*	0.78639	.001	-5.0598	-1.1602
	GROUP C	GROUP A	-10.10000*	0.78639	.000	-12.0498	-8.1502
		GROUP B	3.11000*	0.78639	.001	1.1602	5.0598

The null hypothesis, which is supported by these data, claims that there is no discernible variation in the tear strength of three distinct materials across various time intervals. We can reject the null hypothesis and come to the conclusion that there is a significant change in the tear strength of these gingival mask materials at different time intervals because the p-value is less than the significance level of 0.05.

In the present study, comparison of tensile strength of Group A, Group B, and Group C at time intervals of one day, three days, and seven days was done by one-way ANOVA and Tukey Post Hoc test. Based on results we can say that tensile strength decreases as time increases (Tables 3, 4).

**Table 3:** Comparison of tensile strength between groups and within groups by one-way ANOVA df: Degree of freedom; S: Significant

		Sum of squares	df	Mean Square	F	ANOVA P Value	Significance
Day 1	Between Groups	18.9713	2	9.4857	163.1457	<0.00001	S
	Within Groups	1.5698	27	0.0587			
	Total	20.5411	29				
Day 3	Between Groups	12.8007	2	6.4003	36.04242	<0.00001	S
	Within Groups	4.7946	27	0.1776			
	Total	17.5953	29				
Day 7	Between Groups	29.4958	2	14.77479	229.85567	<0.00001	S
	Within Groups	1.7324	27	0.0642			
	Total	31.2281	29				

**Table 4:** Summary of Tukey Post Hoc test to compare tensile strength of three gingival mask materials with each other at different time intervals *. The mean difference is significant at the 0.05 level.

	(I) Group	(J) Group	Mean Difference (I-J)	Standard Error	Significance	95% Confidence Interval	
						Lower Bound	Upper Bound
Day 1	GROUP A	GROUP B	1.82400^*^	.10784	.000	1.5566	2.0914
		GROUP C	.32000^*^	.10784	.017	.0526	.5874
	GROUP B	GROUP A	-1.82400^*^	.10784	.000	-2.0914	-1.5566
		GROUP C	-1.50400^*^	.10784	.000	-1.7714	-1.2366
	GROUP C	GROUP A	-.32000^*^	.10784	.017	-.5874	-.0526
		GROUP B	1.50400^*^	.10784	.000	1.2366	1.7714
Day 3	GROUP A	GROUP B	1.36000*	.18846	.000	.8927	1.8273
		GROUP C	-.05000	.18846	.962	-.5173	.4173
	GROUP B	GROUP A	-1.36000*	.18846	.000	-1.8273	-.8927
		GROUP C	-1.41000*	.18846	.000	-1.8773	-.9427
	GROUP C	GROUP A	.05000	.18846	.962	-.4173	.5173
		GROUP B	1.41000*	.18846	.000	.9427	1.8773
Day 7	GROUP A	GROUP B	2.29200*	.11328	.000	2.0111	2.5729
		GROUP C	.45000*	.11328	.001	.1691	.7309
	GROUP B	GROUP A	-2.29200*	.11328	.000	-2.5729	-2.0111
		GROUP C	-1.84200*	.11328	.000	-2.1229	-1.5611
	GROUP C	GROUP A	-.45000*	.11328	.001	-.7309	-.1691
		GROUP B	1.84200*	.11328	.000	1.5611	2.1229

The null hypothesis, which is supported by these data, claims that there is no discernible variation in the tensile strength of three distinct materials across various time intervals. We can reject the null hypothesis and come to the conclusion that there is a significant change in the tensile strength of these gingival mask materials at different time intervals because the p-value is less than the significance level of 0.05. We can conclude from the Tukey Post Hoc test that on the third day, Groups A and C were statistically insignificant.

In statistics, the word correlation refers to the relationship between two variables, which here are tear and tensile strengths (Table 5).

**Table 5:** Correlation between tear and tensile strength **. Correlation is significant at the 0.01 level (2-tailed).

		Tensile Strength	Tear Strength
Tensile Strength	Pearson Correlation	1	.540^**^
	Sig. (2-Tailed)		.000
	N	270	270

The correlation coefficient magnitude is 0.54 indicating variables that can be considered moderately correlated. After that linear regression analysis was done to estimate the effect of tensile strength on the tear strength (Tables 6, 7).

**Table 6:** Testing the overall significance of the regression model by ANOVA a. Dependent variable: tear strength;  b. Linear regression through the origin; c. Predictors: tensile strength; d. This total sum of squares is not corrected for the constant because the constant is zero for regression through the origin. df: Degree of freedom

ANOVA^a,b^						
Model		Sum of Squares	df	Mean Square	F	Sig.
1	Regression	5430.351	1	5430.351	328.978	0.00001^c^
	Residual	1469.1	89	16.507		
	Total	6899.451^d^	90			

The p-value is 0.00001, which is less than the common significance level of 0.05. This indicates that the regression model as a whole is statistically significant.

**Table 7:** Coefficient estimates, standard error of the estimates, the t-stat, and p-values in the regression model a. Dependent variable: tear strength; b. Linear regression through the origin

Coefficients^a,b^						
Model		Unstandardized Coefficients		Standardized Coefficients	t	Sig.
		B	Std. Error	Beta		
1	Tensile Strength	2.833	0.156	0.887	18.138	0.00001

The coefficients give us the numbers necessary to write the estimated regression equation:

 Tear strength = 2.833 (Tensile strength)

## Discussion

Many studies in the literature have confirmed the effectiveness of implant-supported treatments, which are often used for both full and partial tooth loss in modern dentistry practices [7,8]. An implant-supported prosthesis needs to meet various criteria to be successful. Among these, passive fit is one of the most important factors that affects the restoration's long-term success [9]. Despite studies evaluating implant-level impression techniques' accuracy, there remains controversy regarding the minimal values necessary to attain passive fit [10]. 

Implant-supported prostheses that fail to achieve passive fit run the risk of developing biological and mechanical difficulties in the peri-implant tissues and repair. The long-term success of implant-supported prostheses can be impacted by biological complications such as bone loss in the peri-implant tissues caused by plaque accumulation, which disrupts the marginal fit and causes mechanical complications like loosening, wear, or fracture of implant components. To create optimal implant-supported restorations, accurate working models must be created to precisely translate the intraoral conditions to the laboratory, to achieve these various implant impression techniques have been developed. The first stage in reducing discrepancies is to select the appropriate imprint technique, which should satisfy the needs of ease of application, minimal working time, and guarantee the creation of accurate models. To determine the emerging profile in fixed restoration, some studies use combination methods that incorporate temporary restoration and scanning [11-14].

A harmonious interaction with the surrounding gingival tissue is another crucial element for the long-term effectiveness of implant-supported prostheses. Oral hygiene practices, the angle at which the prosthesis borders meet the free gingiva surrounding the implant, and the texture of the surfaces that come into touch with the tissue all have an impact on this harmony [15-17]. Gingival infections, hyperplasia, and food impaction are all exacerbated by over-contoured restorations [14]. From this angle, it becomes imperative to precisely transmit the relationship between the gingiva's emerging profile and the restoration of the soft tissue in the pontic zones. It is essential to replicate the improved interaction between the restoration and soft tissue in the manufacturing models to guarantee the longevity of the restoration and satisfy esthetic and phonetic expectations. The intrinsic rigidity of conventional plaster models presents difficulties in designing restorations that blend in harmoniously with the surrounding tissues. Because of its physical characteristics, plaster may distort during the fabrication process, making it unable to precisely replicate the suppleness and durability of actual soft tissues. Therefore, during the restoration's production phase, it becomes crucial to use materials that replicate the free gingiva on the working model.

In 2023, Ak and Bilgin did one study to examine the effects on model accuracy of various gingival masks and production methods [5]. Using the open tray technique, impressions were obtained from an upper jaw model with six parallel implants. Next, three distinct gum masks (Gingifast Elastic, Zhermack, Marl, Germany; Gingifast Rigid, Zhermack; Gi-Mask, Coltene, Altstätten, Switzerland) were used to create 20 models; these were then separated into groups based on whether they were single- or double-piece shapes. Lab scanner was used to scan the generated models. The average angular deviation values for each model were computed after scanned data were examined to determine the angular changes of each analog. The six groups were contrasted with one another. The Kruskal-Wallis test was employed to assess the degree of significance. In comparison to samples produced with condensation silicone and softer types of extra silicone, it was found that samples made with tougher silicone exhibited greater angular deviation. Whether using a single piece or two pieces, the fabrication method did not affect the accuracy of the model. Based on this investigation, it was found that tougher silicone gingival masks were easier to use and manipulate, while they displayed greater angular deviation when compared to the other study groups.

The literature is replete with studies comparing the tear and tensile strength of elastomeric impression materials, but there are currently no published studies on the pros and cons of different gingival masks. The gingival mask is a copy of the peri-implant tissue that is used to provide an optimum restoration. Losing the gingival mask is a clinical issue that hinders the process of fabricating the prosthesis [18]. Thus, the purpose of this work was to quantify the tensile strength and tear strength of several gingival mask materials. Three distinct materials were used in our study as the gingival mask material: an aesthetic mask containing A-silicon, a Gi-mask with C-silicon, and a Gingi fast Rigid which contains vinylpolysiloxanes. Typically, we use these materials to replicate soft tissue during the manufacture of implant prostheses.

Please note the following limitations of the study: The samples were fabricated as per the ASTM D624 and D142 specifications, but thickness and shape varied in each case which might affect results. The specimens used in the investigation were created using a standardized stainless-steel die, which does not mimic the activity of oral tissues. Furthermore, unlike an actual imprint of the tissues, the test specimens were not contained in a mold and their shape did not mirror the impression of the oral tissues.

In the future, it would be beneficial to conduct additional studies to examine the tear strength and tensile strength of materials with varying thickness. Furthermore, it would be valuable to expand this study to compare other properties of gingival mask materials.

## Conclusions

Within the limitations of the laboratory testing condition and materials used for the present study following conclusion are drawn. It is mandatory to choose a material not only depending on the clinical situation but also based on the time taken for the articulation. Tear strength and tensile strength of three gingival mask materials are influenced by the time factor.

As time increases tear strength and tensile strength decrease except Group A (esthetic mask auto mix). In Group A tear strength increases as time increases. Group A (esthetic mask auto mix) has a high tear strength compared to Group B and C (gingifast rigid). Group B (Gi-Mask) has the least tear strength among all three. Tensile strength decreases as time increases but among these three Group A has high strength compared to Groups B and C.
